# Computer-Aided Diagnosis of Gastrointestinal Ulcer and Hemorrhage Using Wireless Capsule Endoscopy: Systematic Review and Diagnostic Test Accuracy Meta-analysis

**DOI:** 10.2196/33267

**Published:** 2021-12-14

**Authors:** Chang Seok Bang, Jae Jun Lee, Gwang Ho Baik

**Affiliations:** 1 Department of Internal Medicine Hallym University College of Medicine Chuncheon Republic of Korea; 2 Institute for Liver and Digestive Diseases Hallym University Chuncheon Republic of Korea; 3 Institute of New Frontier Research Hallym University College of Medicine Chuncheon Republic of Korea; 4 Division of Big Data and Artificial Intelligence Chuncheon Sacred Heart Hospital Chuncheon Republic of Korea; 5 Department of Anesthesiology and Pain Medicine Hallym University College of Medicine Chuncheon Republic of Korea

**Keywords:** artificial intelligence, computer-aided diagnosis, capsule endoscopy, ulcer, hemorrhage, gastrointestinal, endoscopy, review, accuracy, meta-analysis, diagnostic, performance, machine learning, prediction models

## Abstract

**Background:**

Interpretation of capsule endoscopy images or movies is operator-dependent and time-consuming. As a result, computer-aided diagnosis (CAD) has been applied to enhance the efficacy and accuracy of the review process. Two previous meta-analyses reported the diagnostic performance of CAD models for gastrointestinal ulcers or hemorrhage in capsule endoscopy. However, insufficient systematic reviews have been conducted, which cannot determine the real diagnostic validity of CAD models.

**Objective:**

To evaluate the diagnostic test accuracy of CAD models for gastrointestinal ulcers or hemorrhage using wireless capsule endoscopic images.

**Methods:**

We conducted core databases searching for studies based on CAD models for the diagnosis of ulcers or hemorrhage using capsule endoscopy and presenting data on diagnostic performance. Systematic review and diagnostic test accuracy meta-analysis were performed.

**Results:**

Overall, 39 studies were included. The pooled area under the curve, sensitivity, specificity, and diagnostic odds ratio of CAD models for the diagnosis of ulcers (or erosions) were .97 (95% confidence interval, .95–.98), .93 (.89–.95), .92 (.89–.94), and 138 (79–243), respectively. The pooled area under the curve, sensitivity, specificity, and diagnostic odds ratio of CAD models for the diagnosis of hemorrhage (or angioectasia) were .99 (.98–.99), .96 (.94–0.97), .97 (.95–.99), and 888 (343–2303), respectively. Subgroup analyses showed robust results. Meta-regression showed that published year, number of training images, and target disease (ulcers vs erosions, hemorrhage vs angioectasia) was found to be the source of heterogeneity. No publication bias was detected.

**Conclusions:**

CAD models showed high performance for the optical diagnosis of gastrointestinal ulcer and hemorrhage in wireless capsule endoscopy.

## Introduction

Wireless capsule endoscopy (WCE) allows the investigation of gastrointestinal mucosal lesions in a noninvasive manner. This provides approximately 50,000 to 60,000 video frames and allows visualization of the entire gastrointestinal mucosa in a single examination without causing discomfort to patients or risk of procedure-related adverse events [1,2]. Given that the small intestine has been a blind spot for gastroenterologists, WCE has become the standard investigation modality for obscure gastrointestinal hemorrhage and a widely accepted method for the assessment of small intestinal ulcers or tumors [1]. Despite easy accessibility, safety, and patients’ comfort for the examination, WCE has a limitation regarding the interpretation. A tedious reading time of approximately 30 to 120 minutes is required, and a small number of abnormal video frames can be easily mistaken for a normal mucosa by endoscopists [1-3].

Artificial intelligence technology has been adopted in gastrointestinal endoscopy, and the automatic detection or diagnosis of abnormal lesions on endoscopic images or movies has been widely investigated [4,5]. The main benefit of the application of artificial intelligence would be the reduction of the laborious reading time and miss rate of important findings in WCE. Another advantage would be the highly accurate diagnostic performance, which is comparable to that of an endoscopist [6]. These artificial intelligence models are expected to aid in the automatic detection of important lesions in WCE images, thus making it possible to perform automatic reading and interpretation of the entire examination.

Previous studies have reported the performance of computer-aided diagnosis (CAD) models using artificial intelligence in WCE [7,8]. Machine learning– or deep learning–based artificial intelligence models with potential benefits have been reported in these studies. Based on these findings, 2 meta-analyses have been conducted for the pooled diagnostic performance of deep-learning models or convolutional neural network models for the diagnosis of gastrointestinal hemorrhage or ulcers using WCE [7,8]. However, the first meta-analysis searched only 1 database, and a substantial number of important articles were omitted. Moreover, an inaccurate crude number of true positives (TP), false positives (FP), false negatives (FN), or true negatives (TN) of CAD models in each study was reported [7]. This inaccurate pooled diagnostic performance can mislead the readers. The second meta-analysis searched multiple databases; however, it also did not include several important papers, and only a single medical librarian searched all the databases [8]. The main pitfall was the simple pooling of the sensitivity or specificity in each study without considering the distribution of abnormal lesions among the total included lesions in each study. Moreover, the diagnostic performance for the gastrointestinal ulcers and hemorrhage was not separated but combined into a single outcome, and quality assessments in each included study were also omitted. The method of exploring the reason for the heterogeneity and the assessment of publication bias also adhered to the interventional meta-analysis methodology in both meta-analyses but did not satisfy the diagnostic test accuracy (DTA) meta-analysis methodology. Given that the method of conducting interventional and DTA meta-analyses is different and that a widely accepted standard methodology exists in conducting the DTA meta-analysis, this can also mislead the readers (Table 1). Therefore, systematic reviews conducted thus far have been inadequate, and the real diagnostic validity of CAD models in WCE has not yet been determined. This study aimed to evaluate the DTA of CAD models for gastrointestinal ulcers or hemorrhage using WCE images through the standard methodology.

**Table 1:** Comparison of previous meta-analyses with the current study.

Parameters	This study	Soffer et al [7]	Mohan et al [8]
Number of included studies	20 studies on gastrointestinal ulcers and 19 studies on gastrointestinal hemorrhage	5 studies on gastrointestinal ulcers and 5 studies on gastrointestinal hemorrhage	9 studies for the diagnosis of gastrointestinal ulcers or hemorrhage (did not perform separate analysis between ulcers and hemorrhage)
Main outcome	Separate diagnostic performance of CAD^a^ models for the gastrointestinal ulcers or hemorrhage using WCE^b^	Separate diagnostic performance of CAD models for the gastrointestinal ulcers or hemorrhage using WCE	Pooled diagnostic performance of CAD models for gastrointestinal ulcers and hemorrhage using WCE (not a meta-analysis with DTA^c^; lack of consideration for the prevalence of ulcers or hemorrhage in each study and thus no calculation of TP^d^, FP^e^, FN^f^, or TN^g^ in each study)
Search strategy	Search of MEDLINE through PubMed, Web of Science, and the Cochrane Library (2 independent authors searched the databases)	Search of MEDLINE through PubMed (2 independent authors searched the database)	Search of ClinicalTrials.gov, Ovid EBM^h^ Reviews, Ovid, Embase, Ovid MEDLINE, Scopus, and Web of Science (a single medical librarian searched all the databases)
Inaccurate calculation (coding) of TP/FP/FN/TN	N/A^i^	Inaccurate calculation detected in the study’s figures	Not a meta-analysis with DTA; lack of consideration for the prevalence of ulcers or hemorrhage in each study and thus no calculation of TP, FP, FN, or TN in each study
Determination of the heterogeneity between studies	Correlation coefficient between the logarithm of the sensitivity and specificity, beta of HSROC^j^ model, visual examination of the SROC curve	*I*^2^ statistics (DTA meta-analysis did not determine heterogeneity with *I*^2^ statistic)	*I*^2^ statistic (DTA meta-analysis did not determine heterogeneity with *I*^2^ statistics)
Quality assessment	QUADAS-2^k^	QUADAS-2	Not assessed
Publication bias	Deeks funnel plot asymmetry test	Not assessed	Not assessed

^a^CAD: computer-aided diagnosis.

^b^WCE: wireless capsule endoscopy.

^c^DTA: diagnostic test accuracy.

^d^TP: true positive.

^e^FP: false positive.

^f^FN: false negative.

^g^TN: true negative.

^h^EBM: evidence-based medicine.

^i^N/A: not applicable.

^j^HSROC: hierarchical summary receiver operating characteristic.

^k^QUADAS-2: Quality Assessment of Diagnostic Accuracy Studies second version.

## Methods

### Adherence to the Checklist for Systematic Reviews and Meta-analyses

This study was conducted in accordance with the statement of the PRISMA (Preferred Reporting Items for a Systematic Review and Meta-analysis) of DTA Studies [9]. The study protocol was registered at the International Prospective Register of Systematic Reviews (PROSPERO) database before initiation of the systematic review (#CRD42021253454). Approval from the institutional review board of the Chuncheon Sacred Heart Hospital was waived.

### Search Strategy for Relevant Literature

The authors established searching formulae using keywords related to the performance of CAD models in the detection of ulcer or hemorrhage using WCE images. Medical Subject Headings (MeSH) terminology keywords were used for the establishment of searching formulae (Textbox 1).

**Literature searching strategy for the core databases.**

**1. CAD of gastrointestinal ulcers in WCE**

- Database: MEDLINE (through PubMed)

#1. “artificial intelligence”[tiab] OR “AI”[tiab] OR “deep learning”[tiab] OR “machine learning”[tiab] OR “computer”[tiab] OR “neural network”[tiab] OR “CNN”[tiab] OR “automatic”[tiab] OR “automated”[tiab]: 532189

#2. “capsule endoscopy”[tiab] OR “capsule endoscopy”[Mesh]: 5110

#3. “ulcer”[tiab] OR “ulcer”[Mesh] OR “erosion”[tiab]: 138857

#4. #1 AND #2 AND #3: 29

#5. #4 AND English[Lang]: 29

- Database: Web of Science

#1. artificial intelligence OR AI OR deep learning OR machine learning OR computer OR neural network OR CNN OR automatic OR automated: 1236876

#2. capsule endoscopy: 3524

#3. ulcer: 33664

#4. #1 AND #2 AND #3: 49

- Database: Cochrane Library

#1. artificial intelligence: ab, ti, kw; OR AI: ab, ti, kw; OR deep learning: ab, ti, kw; OR machine learning: ab, ti, kw; OR computer: ab, ti, kw; OR neural network: ab, ti, kw; OR CNN: ab, ti, kw; OR automatic: ab, ti, kw; OR automated: ab, ti, kw: 60327

#2. MeSH descriptor: [capsule endoscopy] explode all trees: 131

#3. capsule endoscopy: ab, ti, kw: 724

#4. #2 OR #3: 724

#5. MeSH descriptor: [ulcer] explode all trees: 1413

#6. ulcer: ab, ti, kw; OR erosion: ab, ti, kw: 20844

#7. #5 or #6: 20844

#8. #1 and #4 and #7: 2 trials

**2. CAD of Gastrointestinal hemorrhage in WCE**

- Database: MEDLINE (through PubMed)

#1. “artificial intelligence”[tiab] OR “AI”[tiab] OR “deep learning”[tiab] OR “machine learning”[tiab] OR “computer”[tiab] OR “neural network”[tiab] OR “CNN”[tiab] OR “automatic”[tiab] OR “automated”[tiab]: 532189

#2. “capsule endoscopy”[tiab] OR “capsule endoscopy”[Mesh]: 5110

#3. “bleeding”[tiab] OR “hemorrhage”[Mesh] OR “angioectasia”[tiab]: 475519

#4. #1 AND #2 AND #3: 82

#5. #4 AND English[Lang]: 79

- Database: Web of Science

#1. artificial intelligence OR AI OR deep learning OR machine learning OR computer OR neural network OR CNN OR automatic OR automated: 1236876

#2. capsule endoscopy: 3524

#3. bleeding OR hemorrhage OR angioectasia: 146789

#4 #1 AND #2 AND #3: 87

- Database: Cochrane Library

#1. artificial intelligence: ab, ti, kw; OR AI: ab, ti, kw; OR deep learning: ab, ti, kw or machine learning: ab, ti, kw; OR computer: ab, ti, kw; OR neural network: ab, ti, kw; OR CNN: ab, ti, kw; OR automatic: ab, ti, kw; OR automated: ab, ti, kw: 60327

#2. MeSH descriptor: [capsule endoscopy] explode all trees: 131

#3. capsule endoscopy: ab, ti, kw: 724

#4. #2 or #3: 724

#5. MeSH descriptor: [hemorrhage] explode all trees: 14887

#6. bleeding: ab, ti, kw; OR angioectasia: ab, ti, kw: 46708

#7. #5 OR #6: 53831

#8. #1 AND #4 AND #7: 8 (trials)

**Abbreviations**

CAD, computer-aided diagnosis; WCE: wireless capsule endoscopy; tiab: searching code for title and abstract; Mesh: Medical Subject Headings; ab, ti, kw: search code for abstract, title, and keywords; Lang: search code for language; lim: searching code by limiting certain conditions.

Two authors, CSB and JJL, independently performed a core database search of MEDLINE through PubMed, Web of Science, and Cochrane Library using pre-established search formulae from inception to May 2021. Duplicate articles were excluded. The titles and abstracts of all identified articles were reviewed, and irrelevant articles were excluded. Full-text reviews were subsequently performed to determine whether the pre-established inclusion criteria were satisfied in the identified literature. The references of relevant articles were also reviewed to identify any additional studies. Any disagreements of results in the searching process between CSB and JJL were resolved by discussion or consultation with the other author (GHB).

### Inclusion Criteria of the Literature

The literature included in this systematic review met the following inclusion criteria: designed to evaluate the diagnostic performance of CAD models for gastrointestinal ulcers or hemorrhage based on WCE images; presentation of the diagnostic performance of CAD models, including sensitivity, specificity, likelihood ratios, predictive values, or accuracy, which enabled the estimation of TP, FP, FN, and TN values of CAD models; and written in English. The exclusion criteria were as follows: narrative review articles, studies with incomplete data, systematic review or meta-analyses, comments, proceedings with only an abstract, or study protocols. A full publication with PDF files of available proceedings was considered to be a full article. Articles meeting at least 1 of the exclusion criteria were excluded from this study.

### Assessment of Methodological Quality in the Selected Literature

The methodological quality of the included articles was assessed by CSB and JJL using the second version of Quality Assessment of Diagnostic Accuracy Studies (QUADAS-2). This tool comprises 4 domains, including “patient selection,” “index test,” “reference standard,” and “flow and timing,” with the first 3 domains possessing an “applicability” assessment. CSB and JJL assessed each part as having either a high, low, or unclear risk of bias [10].

### Data Extraction in the Selected Literature, Primary Outcomes of This Study, and Additional Analyses

CSB and JJL independently extracted the data from each included article and cross-checked the extracted data. If data were unclear, the corresponding author of the study was contacted by email to obtain insight into the original data set. A descriptive synthesis was made by a systematic review process, and DTA meta-analysis was conducted if the included studies were sufficiently homogenous.

The primary outcomes were the TP, FP, FN, and TN values in each study. For the CAD of gastrointestinal ulcers or hemorrhage using WCE images, the primary outcomes were defined as follows: TP, the number of patients with a positive finding by a CAD model and who had ulcers or hemorrhage as evidenced by WCE images; FP, the number of patients with a positive finding by a CAD model and who did not have ulcers or hemorrhage based on WCE images; FN, the number of patients with a negative finding by a CAD model and who had ulcers or hemorrhage as evidenced by WCE images; and TN, number of patients with a negative finding by a CAD model and who did not have ulcers or hemorrhage based on the WCE images. With these definitions, TP, FP, FN, and TN values were calculated for each included study.

For additional analysis, such as subgroup analysis or meta-regression, the authors extracted the following variables from each included study: published year, geographic origin of the data (ie, Western vs Asian vs public data or unknown), type of CAD models, type of endoscopic images, number of total images included, type of test data sets (internal test vs external test), and the target disease (ulcer vs erosions, hemorrhage, or angioectasias).

### Statistics

The hierarchical summary receiver operating characteristic (HSROC) method was used for the DTA meta-analysis [11]. A forest plot of the sensitivity and specificity and a SROC curve were generated. The level of heterogeneity across the included articles was determined by the correlation coefficient between logit-transformed sensitivity and specificity by the bivariate method [12]; for this, the asymmetry parameter was β, where β=0 corresponds to a symmetric ROC curve in which the diagnostic odds ratio (DOR) does not vary along the curve according to the HSROC method [11]. A positive correlation coefficient and a β value with a significant probability (*P*<.05) indicate heterogeneity between the studies [11,13]. Visual examination of the SROC curve was also performed to identify heterogeneity. Subgroup analysis by univariate meta-regression using the modifiers identified during the systematic review was also performed to identify the reasons for heterogeneity. STATA software version 15.1 (StataCorp), including the packages “metandi” and “midas,” was used for the DTA meta-analysis. Publication bias was evaluated using the Deeks funnel plot asymmetry test. For the subgroup analyses including less than 4 studies, the Moses-Shapiro-Littenberg method [14] was adopted using Meta-DiSc 1.4 (XI Cochrane Colloquium) because the “metandi” and “midas” packages require the inclusion of a minimum of 4 studies for DTA meta-analysis.

## Results

### Study Selection

**Figure 1:**
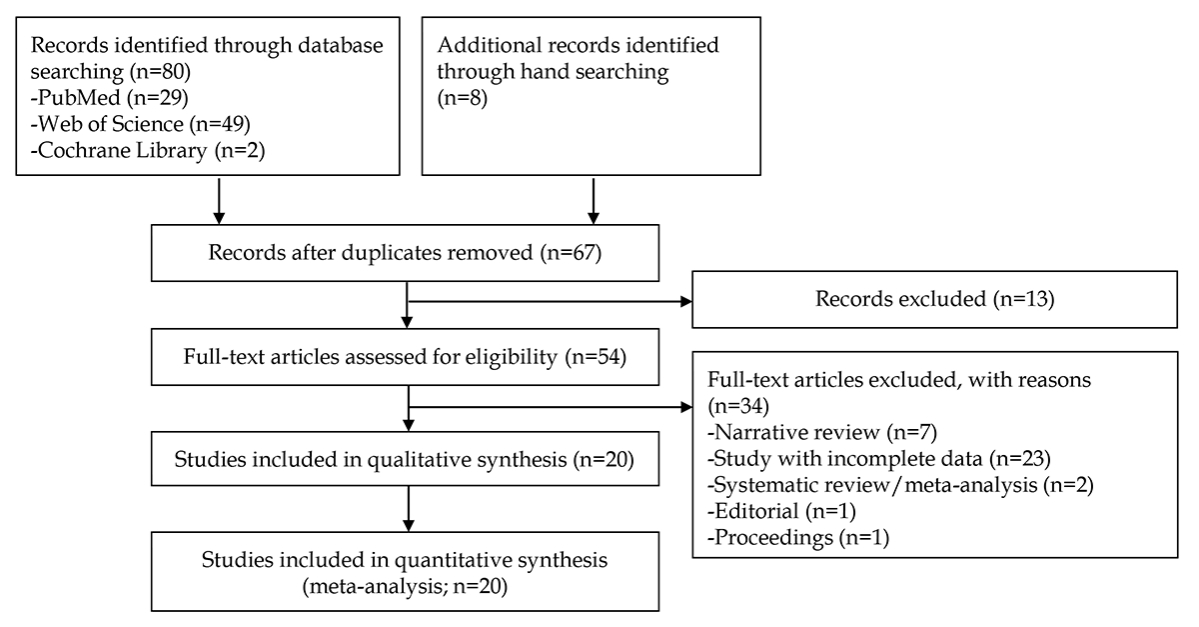
Flowchart of the search process for the diagnostic performance of computer-aided diagnosis for gastrointestinal ulcers or erosions in wireless capsule endoscopy.

**Figure 2:**
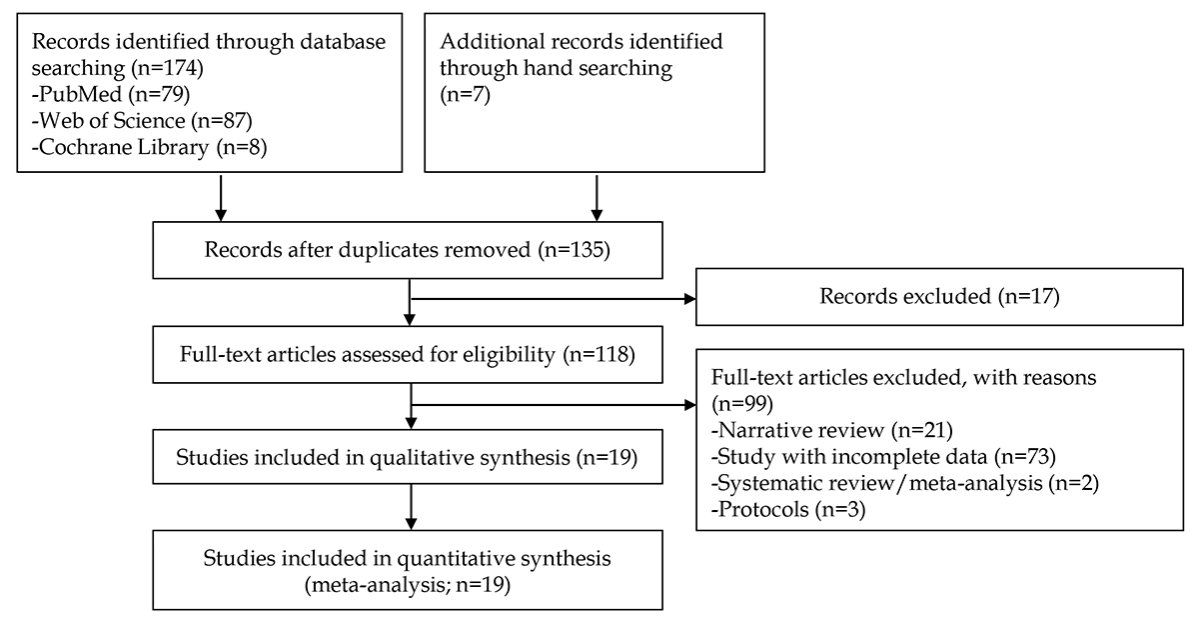
Flowchart of the search process for the diagnostic performance of computer-aided diagnosis for the gastrointestinal hemorrhage in wireless capsule endoscopy.

A total of 254 studies (80 studies for the CAD of gastrointestinal ulcers or erosions and 174 studies for the CAD of gastrointestinal hemorrhage using WCE) were identified following a literature search of 3 databases. Fifteen studies were additionally identified by manual screening of bibliographies. After excluding duplicate studies, additional articles were excluded after a review of titles and abstracts. Full-text versions of the remaining 54 and 118 articles were obtained and thoroughly reviewed based on the aforementioned inclusion and exclusion criteria in each topic. Among these, 133 articles were excluded from the final enrollment. Finally, 20 studies [15-34] for the CAD of gastrointestinal ulcers or erosions and 19 [17,19,24,34-49] studies for the diagnosis of gastrointestinal hemorrhage were included in the systematic review. A flowchart of the selection process is shown in Figure 1 and 2.

### Clinical Features in the Included Studies

Among the 20 studies [15-34] for the CAD of gastrointestinal ulcers or erosions using WCE, a total of 40,809 images were identified (14,866 cases vs 25,943 controls) for the assessment of the diagnostic performance. Given that the duplicate data were identified (Karargyris et al in 2009 [15] and Karargyris et al in 2011 [20]), all the analyses used the data of 19 studies [15-19,21-34] (a study by Karargyris et al in 2011 [20] was omitted in the meta-analysis).

Ten studies [16-18,21,25-27,29,31,32] used endoscopic images from Asian populations, and 3 studies [22,23,33] used endoscopic images from Western populations. However, 6 studies [15,19,24,28,30,34] used public database images or an unknown source of images. Regarding the type of CAD model, a deep neural network or convolutional neural network was used in 9 studies [16-18,27-29,31-33], and machine learning–based models were used in 10 studies [15,19,21-26,30,34]. Most of the included studies [15-28,30-34] presented the diagnostic performance for the intestinal ulcers. However, the study by Aoki et al [29] presented an indistinguishable performance for the intestinal ulcers or erosions, and the study by Fan et al [27] presented a separate performance for the intestinal ulcers and erosions. Therefore, subgroup analyses were performed for the target lesions. Detailed clinical features of the included studies are presented in Multimedia Appendix 1.

Among the 19 studies [17,19,24,34-49] for the diagnosis of gastrointestinal hemorrhage using WCE, a total of 41,323 images were identified (6952 cases vs 34,371 controls) for the assessment of the diagnostic performance.

Five studies [17], [35], [44], [48], [49] used endoscopic images from Asian populations, and 1 study [47] used endoscopic images from Western populations. However, the remaining 13 studies [19,24,34,36-43,45,46] used public database images or an unknown source of images. Regarding the type of CAD model, the deep neural network or convolutional neural network was used in 8 studies [17,35,39,43,46-49], and machine learning–based models were used in 11 studies [19,24,34,36-38,40-42,44,45]. Most of the included studies [17,19,24,34-46,48] presented the diagnostic performance for intestinal hemorrhage. However, studies by Leenhardt et al [47] and Tsuboi et al [49] presented the performance for angiodysplasias. Therefore, subgroup analyses were performed for the target lesions. Detailed clinical features of the included studies are presented in Multimedia Appendix 2.

### Quality Assessment of Study Methodology

The quality and quantity of baseline training data are important because the CAD models are established using learning features of the baseline training data. A sufficient number of training images are required for the establishment of practical CAD models, and endoscopic specialists should participate in the labeling work for the accurate preparation of training data. We also could not guarantee the quality of images in public databases searched on the internet. We determined that proper learning requires at least 30 training images (quantity standard) from real clinical hospital data (quality standard) labeled by an endoscopic specialist (quality standard). If both quality and quantity standards were satisfied, there was considered to be a low risk of bias in the patient selection domain. If only 1 of these quality or quantity standards was satisfied, there was considered to be an unclear risk of bias. If both were not satisfied, there was considered to be a high risk of bias.

**Figure 3:**
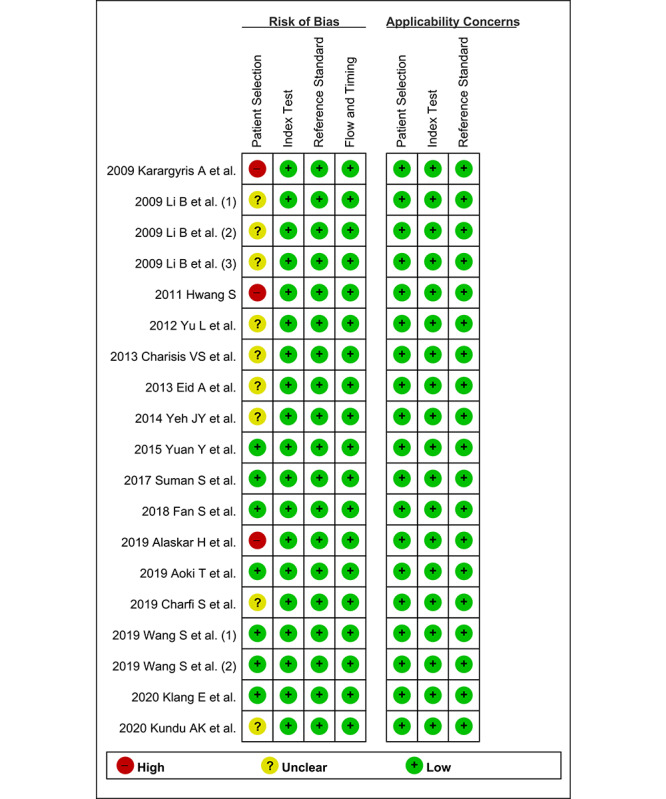
Summary graph of quality in methodology for the computer-aided diagnosis of gastrointestinal ulcers or erosions in wireless capsule endoscopy.

In terms of the CAD of gastrointestinal ulcers or erosions in WCE, only 7 studies [25-27,29,31-34] were rated as low risk of bias, 9 studies [16-18,21-24,30,34] were rated as unclear risk of bias, and 3 studies [15,19,28] were rated as high risk of bias in the “patient selection” domain. The remaining domains were rated as having a low risk of bias in all the included studies (Figure 3). Therefore, classification of methodological quality in the “patient selection” domain was adopted as a modifier in the subgroup or meta-regression analysis.

**Figure 4:**
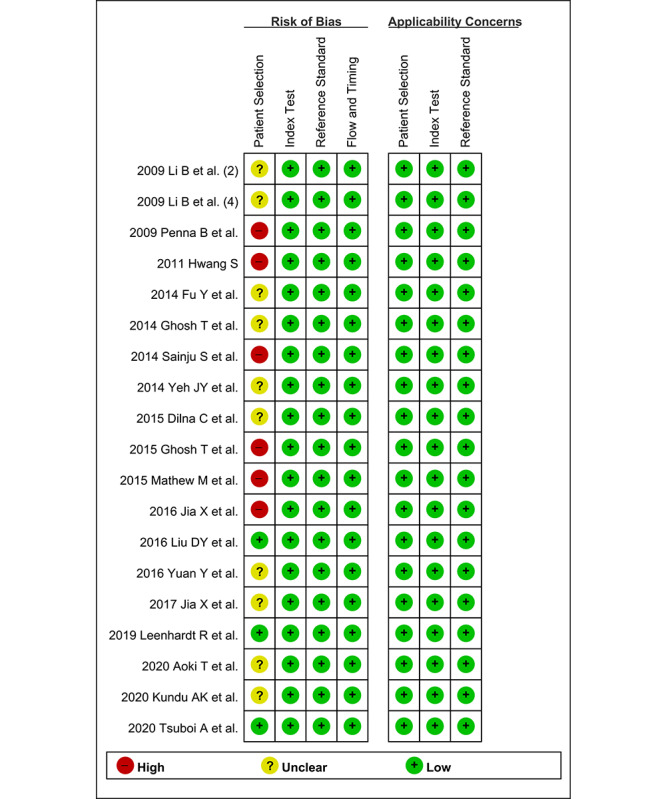
Summary graph of quality in methodology for the computer-aided diagnosis of gastrointestinal hemorrhage in wireless capsule endoscopy.

In terms of the CAD of gastrointestinal hemorrhage in WCE, only 3 studies [44,47,49] were rated as having a low risk of bias, 10 studies [17,24,34,35,37,38,40,45,46,48] were rated as having an unclear risk of bias, and 6 studies [19,36,39,41-43] were rated as having a high risk of bias in the “patient selection” domain. The remaining domains were rated as having a low risk of bias in all the included studies (Figure 4). Therefore, the classification of methodological quality in the “patient selection” domain was adopted as a modifier in the subgroup or meta-regression analysis.

### DTA Meta-analysis for the Performance of CAD Models

**Figure 5:**
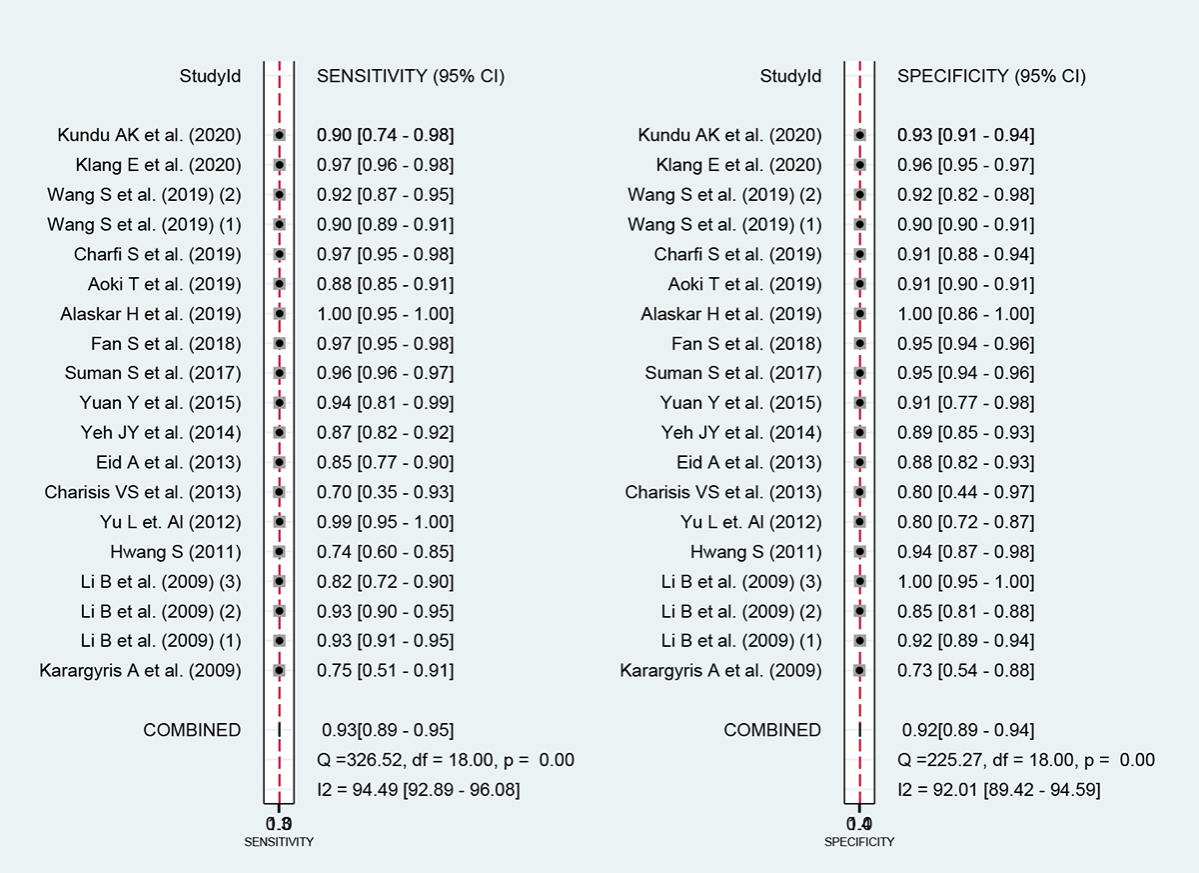
Coupled forest plots of sensitivity and specificity in computer-aided diagnosis models for the diagnosis of gastrointestinal ulcers or erosions in wireless capsule endoscopy images.

**Figure 6:**
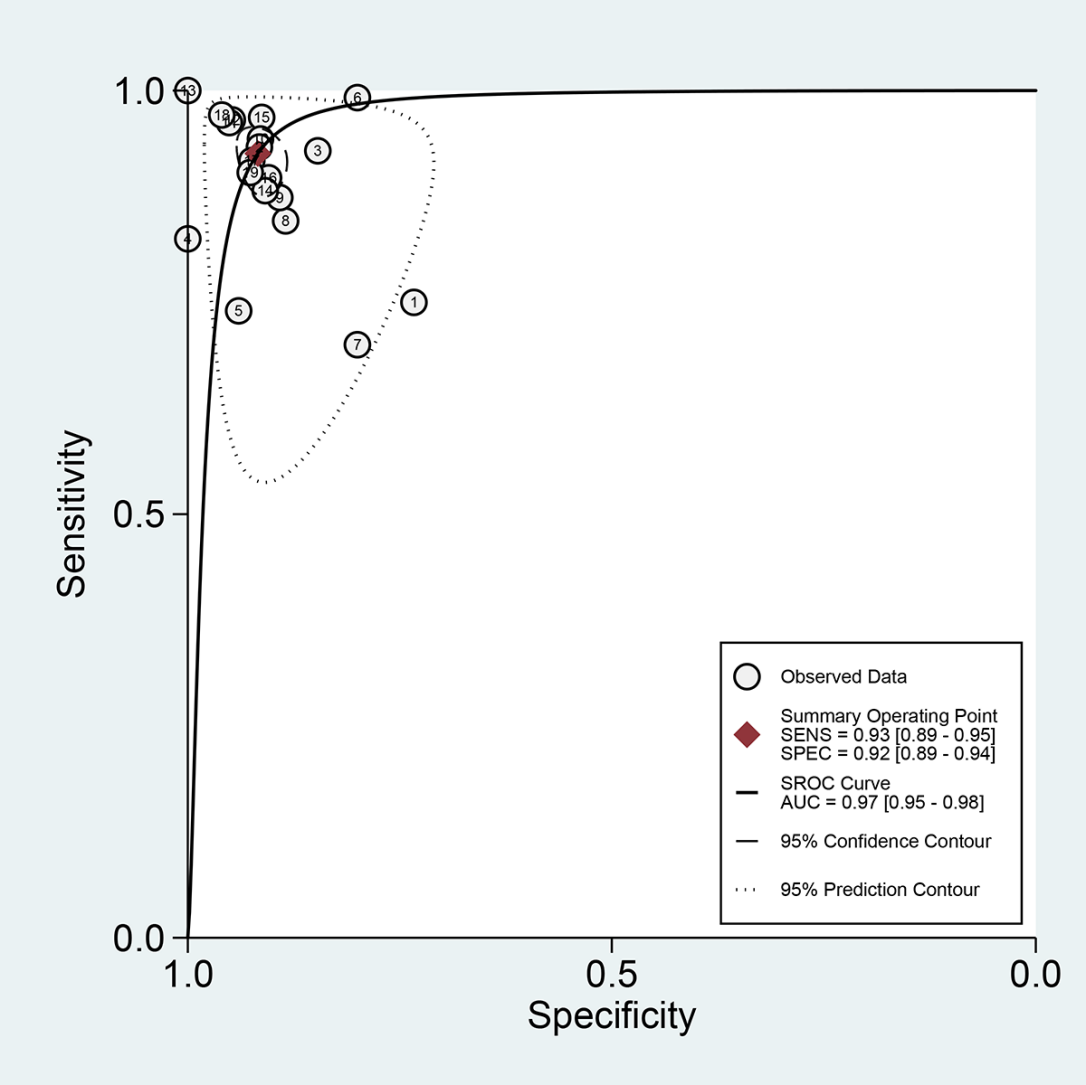
Coupled forest plots of sensitivity and specificity in computer-aided diagnosis models for the diagnosis of gastrointestinal ulcers or erosions in wireless capsule endoscopy images. AUC: area under the curve; SENS: sensitivity; SPEC: specificity; SROC: summary receiver operating characteristic.

**Figure 7:**
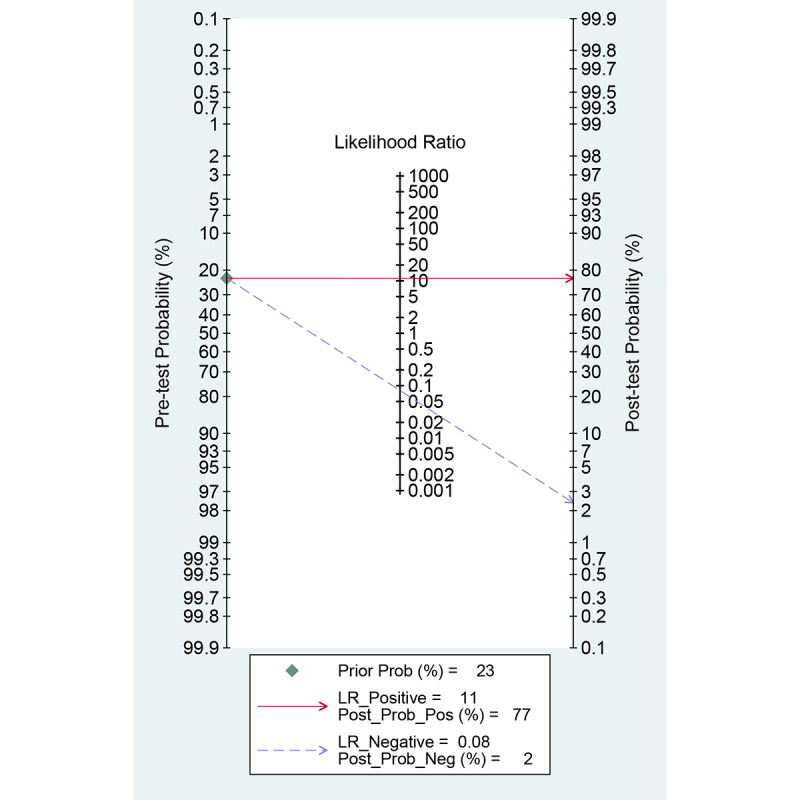
Fagan’s nomogram for the computer-aided diagnosis of gastrointestinal ulcers or erosions in wireless capsule endoscopy images.

Among the 20 studies [15-34] for the meta-analysis of the CAD of gastrointestinal ulcers or erosions using WCE, the area under the curve (AUC), sensitivity, specificity, positive likelihood ratio, negative likelihood ratio, and DOR were 0.97 (95% CI 0.95-0.98), 0.93 (95% CI 0.89-0.95), 0.92 (95% CI 0.89-0.94), 11.2 (95% CI 8.6-14.7), 0.08 (95% CI 0.05-0.12), and 138 (95% CI 79-243), respectively (Multimedia Appendix 3 and Figure 5). The SROC curve is illustrated in Figure 6. To investigate the clinical utility of the CAD models, Fagan’s nomogram [50] was generated. Positive findings indicated that gastrointestinal ulcers or erosions were detected by the CAD models, while negative findings indicated that gastrointestinal ulcers or erosions were not detected by the CAD models. Assuming a 23% prevalence of gastrointestinal ulcers or erosions, Fagan’s nomogram showed that the posterior probability of ulcers or erosions was 76% if the finding of the CAD model was positive and that the posterior probability of ulcers or erosions was only 3% if the finding of the CAD model was negative (Figure 7).

**Figure 8:**
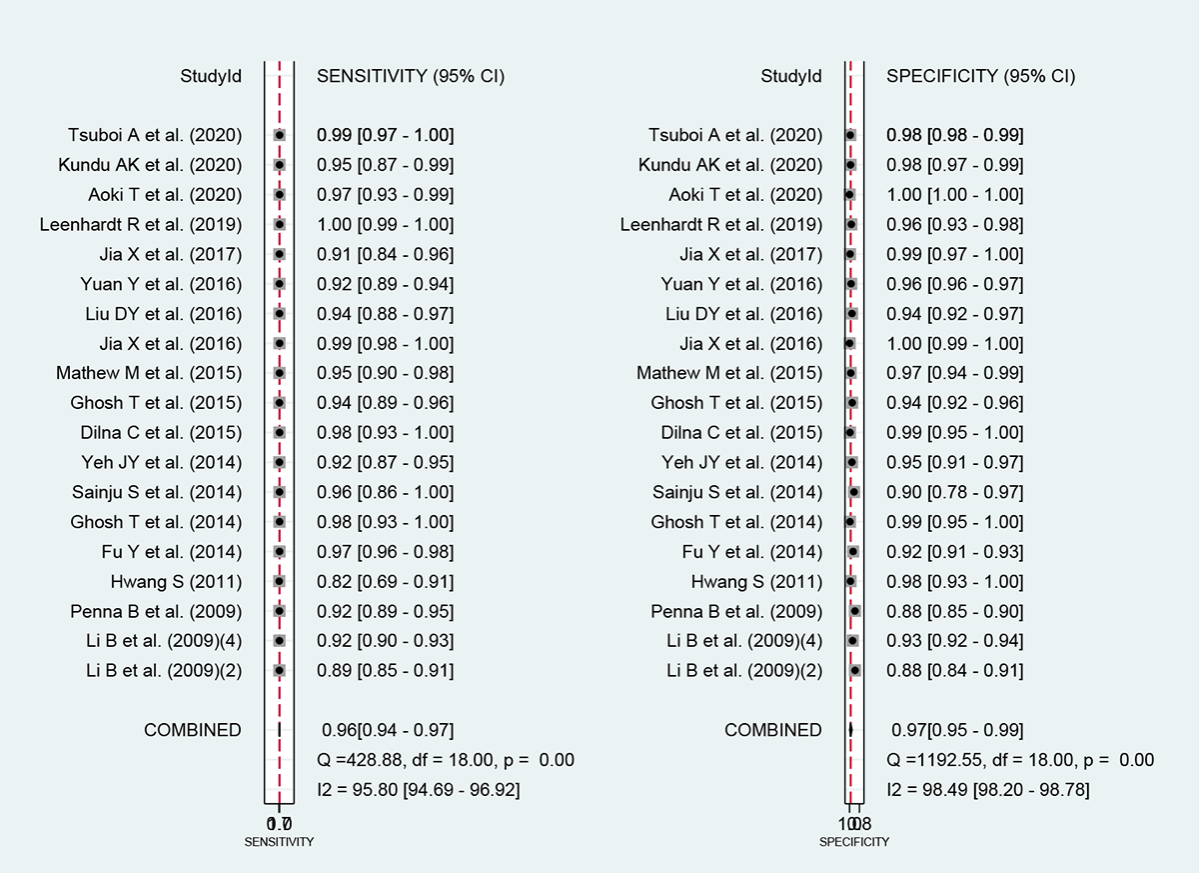
Coupled forest plots of sensitivity and specificity in computer-aided diagnosis models for the diagnosis of gastrointestinal hemorrhage in wireless capsule endoscopy images.

**Figure 9:**
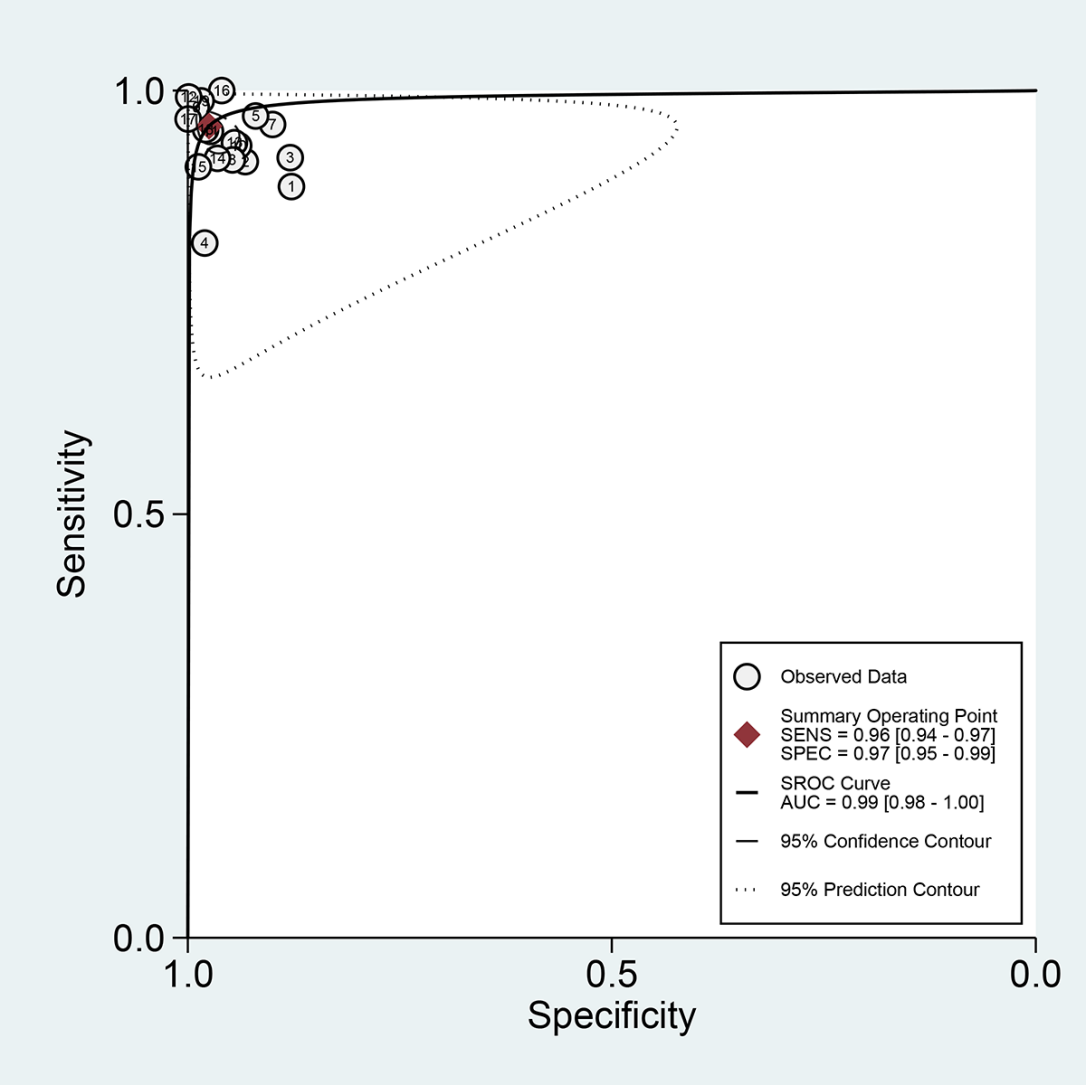
Summary receiver operating characteristic curve with 95% confidence region and prediction region of computer-aided diagnosis models for the diagnosis of gastrointestinal hemorrhage in wireless capsule endoscopy images. AUC: area under the curve; SENS: sensitivity; SPEC: specificity; SROC: summary receiver operating characteristic.

**Figure 10:**
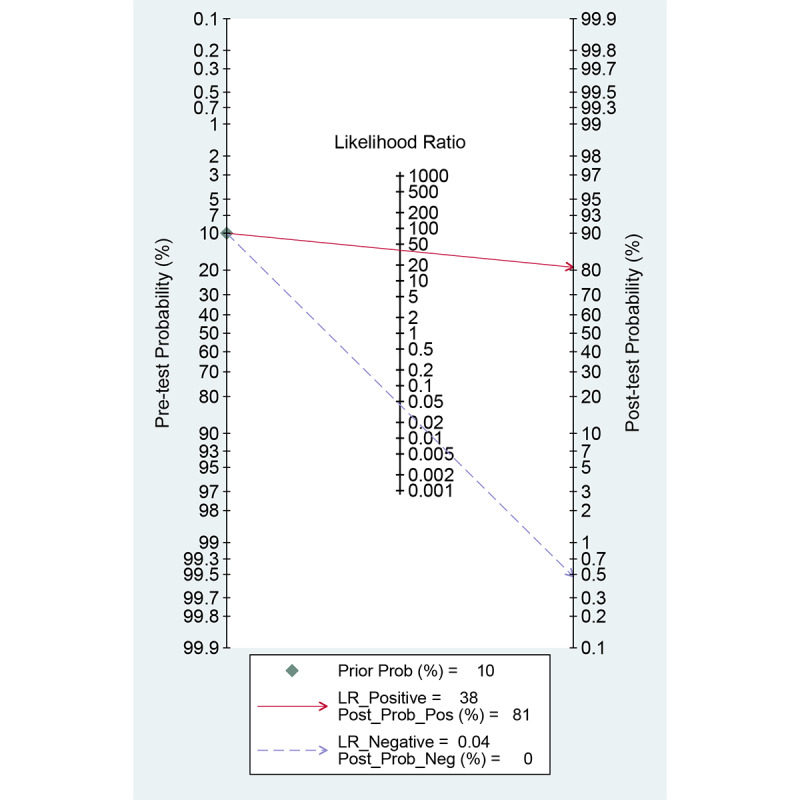
Fagan’s nomogram for the computer-aided diagnosis of small intestinal hemorrhage in wireless capsule endoscopy images.

Among the 19 studies [17,19,24,34-49] for the meta-analysis of the CAD of gastrointestinal hemorrhage in WCE, the AUC, sensitivity, specificity, positive likelihood ratio, negative likelihood ratio, and DOR were 0.99 (95% CI 0.98-0.99), 0.96 (95% CI 0.94-0.97), 0.97 (95% CI 0.95-0.99), 38.3 (95% CI 19.6-74.8), 0.04 (95% CI 0.03-0.07), and 888 (95% CI 343-2303), respectively (Multimedia Appendix 4 and Figure 8). The SROC curve is illustrated in Figure 9. Positive findings of Fagan’s nomogram indicated that gastrointestinal hemorrhage was detected by the CAD models. Negative findings indicated that gastrointestinal hemorrhage was not detected by the CAD models. Assuming a 10% prevalence of small intestinal hemorrhage in all gastrointestinal bleeding [51], Fagan’s nomogram showed that the posterior probability of small intestinal hemorrhage was 81% if the finding of the CAD model was positive and that the posterior probability of small intestinal hemorrhage was only 0.5% if the finding of the CAD model was negative (Figure 10).

### Assessment of Heterogeneity With Meta-regression and Subgroup Analysis

For the CAD of gastrointestinal ulcers or erosions in WCE, we first observed a positive correlation coefficient between the logit-transformed sensitivity and specificity (*r*=0.28) in the bivariate model analysis. However, an asymmetric β parameter in the HSROC model showed an nonsignificant *P* value (*P*=.15), implying that heterogeneity was not present among the studies. Second, a coupled forest plot of sensitivity and specificity was observed (Figure 5). Compared with the enrolled studies, the study by Karargyris et al (2009) [15] showed lower sensitivity and specificity. This study was found to have a high risk of bias in the methodology quality assessment (Figure 3). Therefore, subgroup analysis was carried out according to the methodological quality, and the performance was robust although slightly higher values were observed in the studies of high methodological quality (Multimedia Appendix 3). Third, the shape of the SROC curve for the gastrointestinal ulcers or erosions in WCE was symmetric, and the 95% prediction region was not wide (Figure 6). Fourth, meta-regression using modifiers identified in the systematic review was conducted, and published year, number of training images, and target disease (ulcer vs erosion) were found to be the source of heterogeneity (published year: *P*=.04; number of training images: *P*=.02; target disease ulcer vs erosion: *P*=.38; type of endoscopic image: *P*=.01). Finally, a subgroup analysis based on the potential modifiers was performed, and the overall performance of the studies published within 10 years (vs studies published more than 10 years ago) and studies with more than 100 training images (vs studies with fewer than 100 training images) showed higher values (Multimedia Appendix 3).

For the CAD of gastrointestinal hemorrhage in WCE, we first observed a positive correlation coefficient between the logit-transformed sensitivity and specificity (*r*=0.48) in the bivariate model analysis. However, an asymmetric β parameter in the HSROC model showed an nonsignificant *P* value (*P*=.06), implying that heterogeneity was not present among the studies. Second, a coupled forest plot of sensitivity and specificity was observed (Figure 8), and there was no significant outlier. Third, the shape of the SROC curve for the gastrointestinal ulcers and erosions in WCE was symmetric, and the 95% prediction region was not wide (Figure 9). Fourth, a meta-regression using the modifiers identified in the systematic review was conducted, and published year, number of training images, and target disease (hemorrhage vs angioectasia) were found to be the source of heterogeneity (published year: *P*<.01; number of training images: *P*=.04; target disease hemorrhage vs angioectasia: *P*<.01). Finally, a subgroup analysis based on the potential modifiers was performed, and the overall performance of the studies published within 10 years (vs studies published more than 10 years ago) and studies with more than 100 training images (vs studies with fewer than 100 training images) showed higher values (Multimedia Appendix 4).

### Evaluation of Publication bias

**Figure 11:**
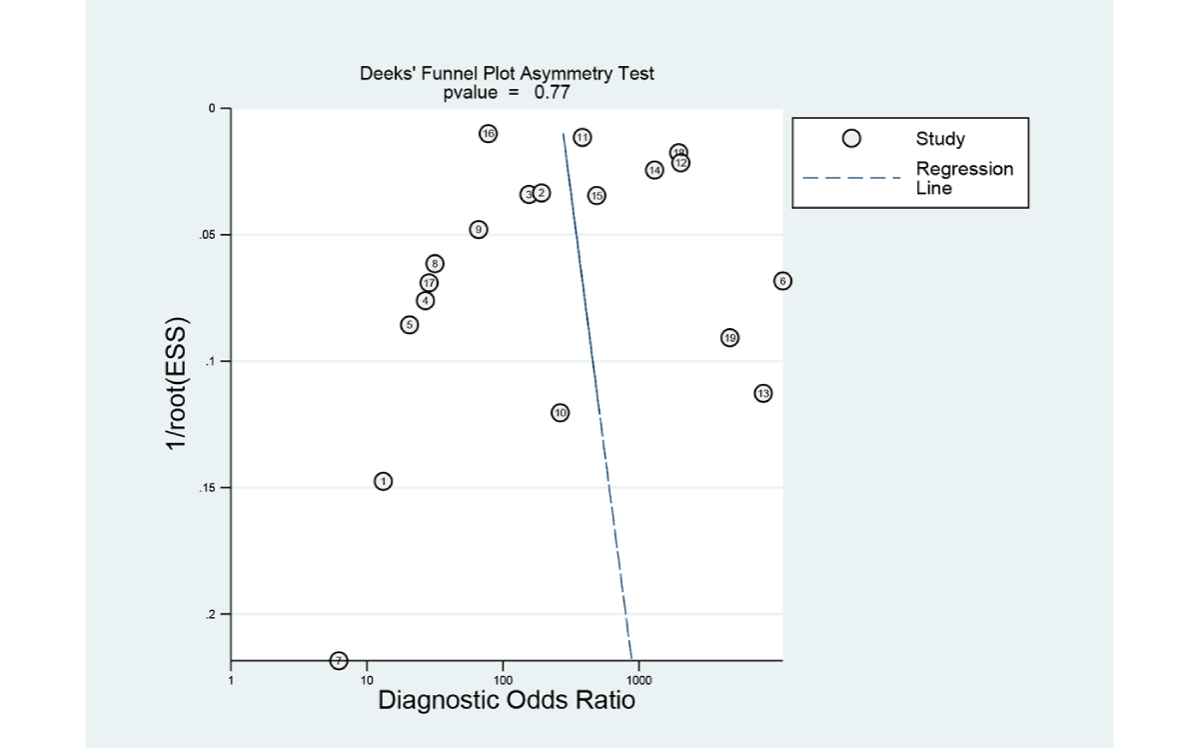
Deeks funnel plot of computer aided diagnosis models for the diagnosis of gastrointestinal ulcers or erosions in wireless capsule endoscopy images.

**Figure 12:**
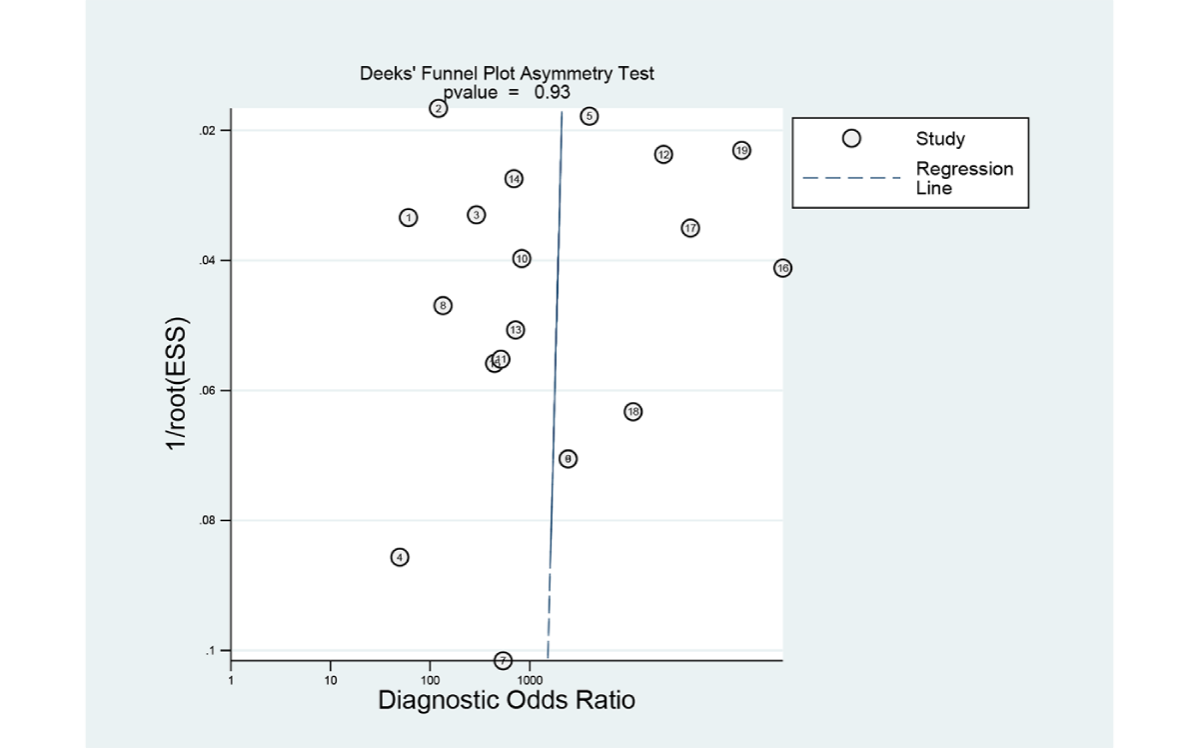
Deeks funnel plot of computer-aided diagnosis models for the diagnosis of gastrointestinal hemorrhage in wireless capsule endoscopy images.

The Deeks funnel plot of studies for the gastrointestinal ulcers or erosions in WCE exhibited a symmetrical shape with respect to the regression line (Figure 11), and the asymmetry test showed no evidence of publication bias (*P*=.77). The Deeks funnel plot of studies for the gastrointestinal hemorrhage in WCE exhibited a symmetrical shape with respect to the regression line (Figure 12), and the asymmetry test showed no evidence of publication bias (*P*=.93).

## Discussion

### Principal Findings

In this study, CAD models showed high performance values for the diagnosis of gastrointestinal ulcers or erosions and hemorrhage in WCE images. Practical values in Fagan’s nomogram indicated the potential to use CAD models in clinical practice. Although the main analyses found some heterogeneity among the included studies, the meta-regression showed the common reasons for heterogeneity (published year, number of training images, and target disease—ulcers vs erosions and hemorrhage vs angioectasia), and subgroup analyses demonstrated that recently published studies (vs studies published more than 10 years ago) with a greater amount of training data (vs studies with fewer than 100 training images) showed better performance of CAD models. Thorough subgroup analyses indicated the robust quality of the evidence.

Interpretation of WCE images is an important task for gastroenterologists. Due to the fact that WCE presents the images of the whole gastrointestinal tract, lesions that are difficult to detect with conventional endoscopy can be identified. Diminutive but important culprit lesions also can be found in the WCE examination. The noninvasive nature of this examination and patients’ comfort have also promoted the use of this technique in the diagnosis of obscure gastrointestinal hemorrhage or small intestinal disorders. However, the interpretation process is tedious. At least 30 to 120 minutes of reading time is required for the endoscopists [1-3]. It is necessary to maintain concentration throughout the reading time so as not to miss important lesions. CAD models have the potential to automate the reading process of WCE with their high diagnostic performance, especially for sensitivity and specificity. The overall performance is slightly higher for gastrointestinal hemorrhage than for ulcers or erosions. It is presumed that this is because red-colored blood is easier to distinguish than are white- or yellow-colored ulcers or erosions, which are similar to the color of the background mucosa for the pixel-based or red-green-blue spectrum–based feature learning of CAD.

In the context of the learning way of CADs, neural network–based CAD models showed a slightly higher performance than that of traditional machine learning–based CAD models (Multimedia Appendices 3 and 4). CNN is not always better than machine learning for accurate classification. However, image recognition with local feature extraction can be highly optimized with its complex layers and deep nodes calculations and dimensional reductions for neural network CAD models. Considering that the machine learning–based models in the included studies used color or textures features in the images of WCE, neural network–based models might focus on the other local features or combined features, such as the shape of the lesions or feature differences between the lesions and background mucosa. Explainable artificial intelligence analyses are on the rise, and the application of this technique would provide a method of determination in the CAD models [52].

Although meta-analyses of same topic have already been published, this study was conducted to evaluate the DTA of CAD models for gastrointestinal ulcers or hemorrhage using WCE images with a standard methodology (Table 1) [7,8]. Although previous studies also reported the high performance of CAD models, many important articles were omitted, the heterogeneity between studies was determined by *I^2^* statistic (which is used in interventional meta-analysis), methodological quality assessments were omitted, and the publication bias was also not assessed.

### Limitations

Despite the robust evidence in this meta-analysis, several inevitable limitations were identified. First, all the performance data were only measured in an internal-test setting in each included study. Modeling is an assumption that observations follow certain statistical rules, and external validation is a method to check whether this assumption is correct or generalizable. Therefore, the confirmation of performance in the established CAD models with unused data in the training or internal testing process is essential [53]. However, no single study conducted performance verification in an external validation setting. Second, the definition of intestinal ulcers or erosions was vague. Erosion usually refers to damage that is limited to the mucosa (loss of the epithelium but with the basement membrane or lamina propria being intact). However, the definition of ulcers usually involves more extensive loss of the mucosa beyond the lamina propria. Although the discrimination between these 2 conditions is not perfect under visual inspection, there was no clear definition in the included studies. This can lead to the underestimation or overestimation of the performance of CAD models. Third, many studies used baseline training data from a public database, and we could not guarantee the quality of images in the public databases available from the internet. The diagnostic performance of the CAD models can only be valid for the population under evaluation and depends on the prevalence of target conditions for the selected population (so-called spectrum bias or class imbalance) [2,54]. This class imbalance was not considered in the included studies. Most of the studies except for 1 [34] applied a 1:1 to 1:4 ratio (target condition:normal mucosa) of the training data set. However, Kundu et al [34] used 31 ulcer images and 1617 normal mucosal images (about a 1:52 ratio) and 65 bleeding images and 1617 normal mucosal images (about a 1:25 ratio) in the training data set. Considering that the method of establishing artificial intelligence models is changing from a model-centric (ie, change or optimize the model to improve performance) to a data-centric approach (ie, systematically change the distribution of the quality of data to improve performance), model establishment that takes into account spectrum bias is required. Overall, qualified training data with clear definitions and a focus on external validation-oriented performance CAD model establishment are required and expected for future perspectives in this topic.

In conclusion, CAD models showed high performance for the optical diagnosis of gastrointestinal ulcers and hemorrhage in WCE.

## Appendix

Multimedia Appendix 1 Clinical characteristics of the included studies for the diagnosis of ulcers or erosions in wireless capsule endoscopy images using computer-aided diagnosis.

Multimedia Appendix 2 Clinical characteristics of the included studies for the diagnosis of gastrointestinal hemorrhage in wireless capsule endoscopy images using computer-aided diagnosis.

Multimedia Appendix 3 Summary of performance and subgroup analysis of the included studies for the diagnosis of ulcers or erosions in wireless capsule endoscopy images using computer-aided diagnosis.

Multimedia Appendix 4 Summary of performance and subgroup analysis of the included studies for the diagnosis of bleeding in wireless capsule endoscopy images using computer-aided diagnosis.

## Abbreviations

AUC: area under the curve
CAD: computer-aided diagnosis
DOR: diagnostic odds ratio
DTA: diagnostic test accuracy
FP: false positive
FN: false negative
HSROC: hierarchical summary receiver operating characteristic
MeSH: Medical Subject Headings
PRISMA: Preferred Reporting Items for a Systematic Review and Meta-analysis
PROSPERO: International Prospective Register of Systematic Reviews
QUADAS-2: the second version of Quality Assessment of Diagnostic Accuracy Studies
TP: true positive
TN: true negative
WCE: wireless capsule endoscopy

## References

[1] Wang A, Banerjee S, Barth BA, Bhat YM, Chauhan S, Gottlieb KT, Konda V, Maple JT, Murad F, Pfau PR, Pleskow DK, Siddiqui UD, Tokar JL, Rodriguez SA, ASGE Technology Committee (2013). Wireless capsule endoscopy. Gastrointest Endosc.

[2] Yang YJ, Bang CS (2019). Application of artificial intelligence in gastroenterology. World J Gastroenterol.

[3] McAlindon ME, Ching H, Yung D, Sidhu R, Koulaouzidis A (2016). Capsule endoscopy of the small bowel. Ann Transl Med.

[4] Bang CS (2020). [Deep learning in upper gastrointestinal disorders: status and future perspectives]. Korean J Gastroenterol.

[5] Bang CS, Lim H, Jeong HM, Hwang SH (2021). Use of endoscopic images in the prediction of submucosal invasion of gastric neoplasms: automated deep learning model development and usability study. J Med Internet Res.

[6] Bang CS, Lee JJ, Baik GH (2021). Computer-aided diagnosis of esophageal cancer and neoplasms in endoscopic images: a systematic review and meta-analysis of diagnostic test accuracy. Gastrointest Endosc.

[7] Soffer S, Klang E, Shimon O, Nachmias N, Eliakim R, Ben-Horin S, Kopylov U, Barash Y (2020). Deep learning for wireless capsule endoscopy: a systematic review and meta-analysis. Gastrointest Endosc.

[8] Mohan BP, Khan SR, Kassab LL, Ponnada S, Chandan S, Ali T, Dulai PS, Adler DG, Kochhar GS (2021). High pooled performance of convolutional neural networks in computer-aided diagnosis of GI ulcers and/or hemorrhage on wireless capsule endoscopy images: a systematic review and meta-analysis. Gastrointest Endosc.

[9] McInnes MDF, Moher D, Thombs BD, McGrath TA, Bossuyt PM, Clifford T, Cohen JF, Deeks JJ, Gatsonis C, Hooft L, Hunt HA, Hyde CJ, Korevaar DA, Leeflang MMG, Macaskill P, Reitsma JB, Rodin R, Rutjes AWS, Salameh J, Stevens A, Takwoingi Y, Tonelli M, Weeks L, Whiting P, Willis BH, the PRISMA-DTA Group (2018). Preferred Reporting Items for a Systematic Review and Meta-Analysis of Diagnostic Test Accuracy Studies: The PRISMA-DTA Statement. JAMA.

[10] Whiting PF, Rutjes AWS, Westwood ME, Mallett S, Deeks JJ, Reitsma JB, Leeflang MMG, Sterne JAC, Bossuyt PMM, QUADAS-2 G (2011). QUADAS-2: a revised tool for the quality assessment of diagnostic accuracy studies. Ann Intern Med.

[11] Rutter CM, Gatsonis CA (2001). A hierarchical regression approach to meta-analysis of diagnostic test accuracy evaluations. Stat Med.

[12] Reitsma JB, Glas AS, Rutjes AWS, Scholten RJPM, Bossuyt PM, Zwinderman AH (2005). Bivariate analysis of sensitivity and specificity produces informative summary measures in diagnostic reviews. J Clin Epidemiol.

[13] Harbord RM, Whiting P (2009). Metandi: meta-analysis of diagnostic accuracy using hierarchical logistic regression. The Stata Journal.

[14] Littenberg B, Moses LE (1993). Estimating diagnostic accuracy from multiple conflicting reports: a new meta-analytic method. Med Decis Making.

[15] Karargyris A, Bourbakis N (2009). Identification of ulcers in wireless capsule endoscopy videos.

[16] Li B, Meng MQ (2009). Texture analysis for ulcer detection in capsule endoscopy images. Image and Vision Computing.

[17] Li B, Meng MQ (2009). Computer-based detection of bleeding and ulcer in wireless capsule endoscopy images by chromaticity moments. Comput Biol Med.

[18] Li B, Qi L, Meng M, Fan Y (2009). Using ensemble classifier for small bowel ulcer detection in wireless capsule endoscopy images.

[19] Hwang S (2011). Bag-of-visual-words approach to abnormal image detection in wireless capsule endoscopy videos.

[20] Karargyris A, Bourbakis N (2011). Detection of small bowel polyps and ulcers in wireless capsule endoscopy videos. IEEE Trans. Biomed. Eng.

[21] Yu L, Yuen PC (2012). Lai J: Ulcer detection in wireless capsule endoscopy images, In Proceedings of the 21st International Conference on Pattern Recognition (ICPR2012), IEEE, 2012 Nov;45-48.

[22] Charisis V, Katsimerou C, Hadjileontiadis L, Liatsos C (2013). Sergiadis GD: Computer-aided capsule endoscopy images evaluation based on color rotation and texture features: An educational tool to physicians, In Proceedings of the 26th IEEE International Symposium On Computer-Based Medical Systems, IEEE, 2013;203-208.

[23] Eid A, Charisis VS, Hadjileontiadis LJ, Sergiadis GD (2013). A curvelet-based lacunarity approach for ulcer detection from wireless capsule endoscopy images, In Proceedings of the 26th IEEE International Symposium on Computer-Based Medical Systems, IEEE, 2013; 273-278.

[24] Yeh J, Wu T, Tsai W (2014). Bleeding and ulcer detection using wireless capsule endoscopy images. JSEA.

[25] Yuan Y, Wang J, Li B, Meng MQ (2015). Saliency based ulcer detection for wireless capsule endoscopy diagnosis. IEEE Trans Med Imaging.

[26] Suman S, Hussin F, Malik A, Ho S, Hilmi I, Leow A, Goh K (2017). Feature selection and classification of ulcerated lesions using statistical analysis for WCE images. Applied Sciences.

[27] Fan S, Xu L, Fan Y, Wei K, Li L (2018). Computer-aided detection of small intestinal ulcer and erosion in wireless capsule endoscopy images. Phys Med Biol.

[28] Alaskar H, Hussain A, Al-Aseem N, Liatsis P, Al-Jumeily D (2019). Application of convolutional neural networks for automated ulcer detection in wireless capsule endoscopy images. Sensors (Basel).

[29] Aoki T, Yamada A, Aoyama K, Saito H, Tsuboi A, Nakada A, Niikura R, Fujishiro M, Oka S, Ishihara S, Matsuda T, Tanaka S, Koike K, Tada T (2019). Automatic detection of erosions and ulcerations in wireless capsule endoscopy images based on a deep convolutional neural network. Gastrointest Endosc.

[30] Charfi S, El Ansari M (2017). Computer-aided diagnosis system for ulcer detection in wireless capsule endoscopy videos, In 2017 International Conference on Advanced Technologies for Signal and Image Processing (ATSIP), IEEE, 2017;1-5.

[31] Wang S, Xing Y, Zhang L, Gao H, Zhang H (2019). A systematic evaluation and optimization of automatic detection of ulcers in wireless capsule endoscopy on a large dataset using deep convolutional neural networks. Phys Med Biol.

[32] Wang S, Xing Y, Zhang L, Gao H, Zhang H (2019). Deep convolutional neural network for ulcer recognition in wireless capsule endoscopy: experimental feasibility and optimization. Comput Math Methods Med.

[33] Klang E, Barash Y, Margalit RY, Soffer S, Shimon O, Albshesh A, Ben-Horin S, Amitai MM, Eliakim R, Kopylov U (2020). Deep learning algorithms for automated detection of Crohn's disease ulcers by video capsule endoscopy. Gastrointest Endosc.

[34] Kundu AK, Fattah SA, Wahid KA (2020). Multiple Linear Discriminant Models for Extracting Salient Characteristic Patterns in Capsule Endoscopy Images for Multi-Disease Detection. IEEE J Transl Eng Health Med.

[35] Li B, Meng MQ (2009). Computer-aided detection of bleeding regions for capsule endoscopy images. IEEE Trans Biomed Eng.

[36] Penna B, Tillo T, Grangetto M, Magli E, Olmo G (2009). A technique for blood detection in wireless capsule endoscopy images.

[37] Fu Y, Zhang W, Mandal M, Meng MQ (2014). Computer-aided bleeding detection in WCE video. IEEE J Biomed Health Inform.

[38] Ghosh T, Bashar S, Alam M, Wahid K, Fattah SA (2014). A statistical feature based novel method to detect bleeding in wireless capsule endoscopy images, IEEE, 2014;1-4.

[39] Sainju S, Bui FM, Wahid KA (2014). Automated bleeding detection in capsule endoscopy videos using statistical features and region growing. J Med Syst.

[40] Dilna C, Gopi V (2015). A novel method for bleeding detection in Wireless Capsule Endoscopic images.

[41] Ghosh T, Fattah S, Bashar S, Shahnaz C, Wahid K, Zhu WP (2015). An automatic bleeding detection technique in wireless capsule endoscopy from region of interest, In.

[42] Mathew M, Gopi VP (2015). Transform based bleeding detection technique for endoscopic images.

[43] Meng MQ, Xiao Jia (2016). A deep convolutional neural network for bleeding detection in Wireless Capsule Endoscopy images. Annu Int Conf IEEE Eng Med Biol Soc.

[44] Liu D, Gan T, Rao N, Xing Y, Zheng J, Li S, Luo C, Zhou Z, Wan Y (2016). Identification of lesion images from gastrointestinal endoscope based on feature extraction of combinational methods with and without learning process. Med Image Anal.

[45] Yuan Y, Li B, Meng MQ (2016). Bleeding Frame and Region Detection in the Wireless Capsule Endoscopy Video. IEEE J Biomed Health Inform.

[46] Meng MQ, Xiao Jia (2017). Gastrointestinal bleeding detection in wireless capsule endoscopy images using handcrafted and CNN features. Annu Int Conf IEEE Eng Med Biol Soc.

[47] Leenhardt R, Vasseur P, Li C, Saurin JC, Rahmi G, Cholet F, Becq A, Marteau P, Histace A, Dray X, CAD-CAP Database Working Group (2019). A neural network algorithm for detection of GI angiectasia during small-bowel capsule endoscopy. Gastrointest Endosc.

[48] Aoki T, Yamada A, Kato Y, Saito H, Tsuboi A, Nakada A, Niikura R, Fujishiro M, Oka S, Ishihara S, Matsuda T, Nakahori M, Tanaka S, Koike K, Tada T (2020). Automatic detection of blood content in capsule endoscopy images based on a deep convolutional neural network. J Gastroenterol Hepatol.

[49] Tsuboi A, Oka S, Aoyama K, Saito H, Aoki T, Yamada A, Matsuda T, Fujishiro M, Ishihara S, Nakahori M, Koike K, Tanaka S, Tada T (2020). Artificial intelligence using a convolutional neural network for automatic detection of small-bowel angioectasia in capsule endoscopy images. Dig Endosc.

[50] Vuik FER, Nieuwenburg SAV, Moen S, Schreuders EH, Oudkerk Pool MD, Peterse EFP, Spada C, Epstein O, Fernández-Urién Ignacio, Hofman A, Kuipers EJ, Spaander MCW (2020). Population-Based Prevalence of Gastrointestinal Abnormalities at Colon Capsule Endoscopy. Clin Gastroenterol Hepatol.

[51] Murphy B, Winter DC, Kavanagh DO (2019). Small Bowel Gastrointestinal Bleeding Diagnosis and Management-A Narrative Review. Front Surg.

[52] Bang CS, Ahn JY, Kim J, Kim Y, Choi IJ, Shin WG (2021). Establishing Machine Learning Models to Predict Curative Resection in Early Gastric Cancer with Undifferentiated Histology: Development and Usability Study. J Med Internet Res.

[53] Bang CS, Lee JJ, Baik GH (2021). Computer-aided diagnosis of diminutive colorectal polyps in endoscopic images: systematic review and meta-analysis of diagnostic test accuracy. J Med Internet Res.

[54] Bang CS, Lee JJ, Baik GH (2020). Artificial intelligence for the prediction of Helicobacter pylori infection in endoscopic images: systematic review and meta-analysis of diagnostic test accuracy. J Med Internet Res.

