# Correction: Computer-Aided Diagnosis of Gastrointestinal Ulcer and Hemorrhage Using Wireless Capsule Endoscopy: Systematic Review and Diagnostic Test Accuracy Meta-analysis

**DOI:** 10.2196/36170

**Published:** 2022-01-11

**Authors:** Chang Seok Bang, Jae Jun Lee, Gwang Ho Baik

**Affiliations:** 1 Department of Internal Medicine Hallym University College of Medicine Chuncheon Republic of Korea; 2 Institute for Liver and Digestive Diseases Hallym University Chuncheon Republic of Korea; 3 Institute of New Frontier Research Hallym University College of Medicine Chuncheon Republic of Korea; 4 Division of Big Data and Artificial Intelligence Chuncheon Sacred Heart Hospital Chuncheon Republic of Korea; 5 Department of Anesthesiology and Pain Medicine Hallym University College of Medicine Chuncheon Republic of Korea

In “Computer-Aided Diagnosis of Gastrointestinal Ulcer and Hemorrhage Using Wireless Capsule Endoscopy: Systematic Review and Diagnostic Test Accuracy Meta-analysis” (J Med Internet Res 2021;23(12):e33267), one error was noted.

In the originally published paper, the Acknowledgments section appeared as follows:

This work was supported by the Technology development Program (#S2931703) funded by the Ministry of SMEs and Startups (Korea).

In the corrected version of the paper, the Acknowledgments section has been revised as follows:

This work was supported by the Institute of Information & Communications Technology Planning & Evaluation (IITP) grant funded by the Korean government (MSIT) (grant number 2020-0-01604).

The correction will appear in the online version of the paper on the JMIR Publications website on January 11, 2022, together with the publication of this correction notice. Because this was made after submission to PubMed, PubMed Central, and other full-text repositories, the corrected article has also been resubmitted to those repositories.

