# Supplementary material for: Computer-Aided Diagnosis of Gastrointestinal Ulcer and Hemorrhage Using Wireless Capsule Endoscopy: Systematic Review and Diagnostic Test Accuracy Meta-analysis
Source: J Med Internet Res. 2021 Dec 14;23(12):e33267. doi: 10.2196/33267 (PMC8715364; doi:10.2196/33267)
Supplement: Multimedia Appendix 1 [file jmir_v23i12e33267_app1.docx]

**Multimedia appendix 1.** Clinical characteristics of the included studies for the diagnosis of ulcers or erosions in wireless capsule endoscopy images using computer-aided diagnosis.

| Study and year | Nationality (data) | Type of CAD^a^ models | Type of endoscopic images | Training data set | Type of test data sets | Number of cases in test data set (ulcers or erosions) | Number of controls in test data set | TP^b^ | FP^c^ | FN^d^ | TN^e^ | Target condition |
| --- | --- | --- | --- | --- | --- | --- | --- | --- | --- | --- | --- | --- |
| Karargyris et al (2009) [15] | Unknown | Texture analysis with SVM^f^ | Still-cut images | 10 ulcer images | Internal test | 20 | 30 | 15 | 8 | 5 | 22 | For ulcer diagnosis (duplicate data) |
| Li et al (2009) [16] | China | Texture analysis with MLP^g^ or SVM | Still-cut images | 1350 ulcer images and 1350 normal mucosal images (from 5 patients) | Internal test | 450 | 450 | 420 | 38 | 30 | 412 | For ulcer diagnosis |
| Li et al (2009) [17] | China | Texture analysis with neural network | Still-cut images | 1350 ulcer images and 1350 normal mucosal images (from 10 patients) | Internal test | 450 | 450 | 418 | 69 | 32 | 381 | For ulcer diagnosis |
| Li et al (2009) [18] | China | Ensemble of SVM, MLP, and kNN^h^ | Still-cut images |  | Internal test | 80 | 80 | 66 | 0 | 14 | 80 | For ulcer diagnosis |
| Hwang (2011) [19] | Unknown | Bow model-SVM | Still-cut images | 25 ulcer images and 50 normal mucosal images | Internal test | 50 | 100 | 37 | 6 | 13 | 94 | For ulcer diagnosis |
| Karargyris et al (2011) [20] | Unknown | Texture analysis with SVM | Still-cut images | 10 ulcer images | Internal test | 20 | 30 | 15 | 8 | 5 | 22 | For ulcer diagnosis (duplicate data) |
| Yu et. Al (2012) [21] | China | Bow model-SVM | Still-cut images | 172 ulcer images and 172 normal mucosal images (from 60 patients) | Internal test | 120 | 120 | 119 | 24 | 1 | 96 | For ulcer diagnosis |
| Charisis et al (2013) [22] | Greece | Texture analysis with SVM | Still-cut images | 100 ulcer images and 100 normal mucosal images (from 6 patients) | Internal test | 10 | 10 | 7 | 2 | 3 | 8 | For ulcer diagnosis |
| Eid et al (2013) [23] | Greece | Texture analysis with SVM | Still-cut images | 130 ulcer images and 130 normal mucosal images | Internal test | 130 | 130 | 110 | 15 | 20 | 115 | For ulcer diagnosis |
| Yeh et al (2014) [24] | Public database | Ensemble of SVM-RFE^i^, neural network, and decision tree | Still-cut images | 190 ulcer images and 258 normal mucosal images | Internal test | 190 | 258 | 166 | 28 | 24 | 230 | For ulcer diagnosis (crude value of highest accuracy) |
|  |  |  |  |  |  |  |  | 167 | 41 | 23 | 217 | For ulcer diagnosis (crude value of highest sensitivity) |
| Yuan et al (2015) [25] | China | Saliency-based analysis with SVM | Still-cut images | 135 ulcer images and 135 normal mucosal images (from 20 patients) | Internal test | 35 | 35 | 33 | 3 | 2 | 32 | For ulcer diagnosis |
| Suman et al (2017) [26] | Malaysia | SVM | Still-cut images | 24000 images (15000 ulcer and 9000 normal images) | Internal test | 5000 | 3000 | 4811 | 147 | 189 | 2853 | For ulcer diagnosis |
| Fan et al (2018) [27] | China | AlexNet | Still-cut images | 2000 ulcer images and 2400 normal mucosal images (from 144 patients) | Internal test | 750 | 2000 | 724 | 105 | 26 | 1895 | For ulcer diagnosis |
|  |  |  |  | 2720 erosion images and 3200 normal mucosal images (from 144 patients) | Internal test | 1500 | 4000 | 1405 | 161 | 95 | 3839 | For erosion diagnosis |
| Alaskar et al (2019) [28] | Database | AlexNet, GoogLeNet | Still-cut images | 256 ulcer images and 80 normal mucosal images | Internal test | 80 | 25 | 80 | 0 | 0 | 25 | For ulcer diagnosis |
| Aoki et al (2019) [29] | Japan | Single Shot multibox Detector | Still-cut images | 5360 images of erosions and ulcerations (from 115 patients) | Internal test | 440 | 10000 | 388 | 913 | 52 | 9087 | For ulcer or erosion diagnosis |
| Charfi et al (2019) [30] | Database | Texture analysis with SVM | Still-cut images | 1400 images (1000 ulcers and 400 normal images) | Internal test | 600 | 333 | 581 | 29 | 19 | 304 | For ulcer diagnosis |
| Wang et al (2019) [31] | China | RetinaNet | Still-cut images | 15871 ulcer images and 17138 normal mucosal images (from 1504 patients) | Internal test | 4917 | 5007 | 4411 | 477 | 506 | 4530 | For ulcer diagnosis |
| Wang et al (2019) [32] | China | ResNet-34 based HAnet | Still-cut images | 990 images (from 1416 patients) | Internal test | 230 | 53 | 211 | 4 | 19 | 49 | For ulcer diagnosis |
| Klang et al (2020) [33] | Israel | XceptionNet | Still-cut images | 6108 ulcer images and 8004 normal mucosal images (from 49 patients) | Internal test | 1283 | 2245 | 1246 | 90 | 37 | 2155 | For Crohn disease ulcer diagnosis |
| Kundu et al (2020) [34] | Database | Linear discriminant analysis with SVM | Still-cut images | 31 ulcer images and 1617 normal mucosal images | Internal test | 31 | 1617 | 28 | 119 | 3 | 1498 | For ulcer diagnosis |

^a^CAD: computer-aided diagnosis.

^b^TP: true positive.

^c^FP: false positive.

^d^FN: false negative.

^e^TN: true negative.

^f^SVM: support vector machine.

^g^MLP: multilayer perceptron.

^h^kNN: k-nearest neighbors algorithm.

^i^RFE: recursive feature elimination.
