# Supplementary material for: Computer-Aided Diagnosis of Gastrointestinal Ulcer and Hemorrhage Using Wireless Capsule Endoscopy: Systematic Review and Diagnostic Test Accuracy Meta-analysis
Source: J Med Internet Res. 2021 Dec 14;23(12):e33267. doi: 10.2196/33267 (PMC8715364; doi:10.2196/33267)
Supplement: Multimedia Appendix 2 [file jmir_v23i12e33267_app2.docx]

**Multimedia appendix 2.** Clinical characteristics of the included studies for the diagnosis of gastrointestinal hemorrhage in wireless capsule endoscopy images using computer-aided diagnosis.

| Study and year | Nationality (data) | Type of CAD^a^ models | Type of endoscopic images | Training data set | Type of test data sets | Number of cases in test data set (ulcers or erosions) | Number of controls in test data set | TP^b^ | FP^c^ | FN^d^ | TN^e^ | Target condition |
| --- | --- | --- | --- | --- | --- | --- | --- | --- | --- | --- | --- | --- |
| Li et al (2009) [17] | China | Texture analysis with neural network | Still-cut images | 1350 ulcer images and 1350 normal mucosal images | Internal test | 450 | 450 | 399 | 55 | 51 | 395 | For bleeding diagnosis |
| Li et al (2009) [35] | China | Texture analysis with neural network | Still-cut images | 1800 ulcer images and 1800 normal mucosal images | Internal test | 1800 | 1800 | 1649 | 122 | 151 | 1678 | For bleeding diagnosis |
| Penna et al (2009) [36] | Public database | Reed-Xiaoli (RX) anomaly detector | Still-cut images |  | Internal test | 341 | 770 | 314 | 93 | 27 | 677 | For bleeding diagnosis |
| Hwang (2011) [19] | Unknown | BoW^f^ model-SVM^g^ | Still-cut images | 25 bleeding images and 50 normal mucosal images | Internal test | 50 | 100 | 41 | 2 | 9 | 98 | For bleeding diagnosis |
| Fu et al (2014) [37] | Public database | Superpixel segmentation (linear clustering with Gaussian filter) with SVM | Still-cut images | 20000 bleeding pixels and 40000 nonbleeding pixels | Internal test | 1000 | 4000 | 970 | 320 | 30 | 3680 | For bleeding diagnosis |
| Ghosh et al (2014) [38] | Public database | Statistical feature detection with kNN^h^ | Still-cut images | 100 bleeding images and 100 normal mucosal images | Internal test | 100 | 100 | 98 | 1 | 2 | 99 | For bleeding diagnosis |
| Sainju et al (2014) [39] | Public database | MLP^i^ | Still-cut images |  | Internal test | 50 | 50 | 48 | 5 | 2 | 45 | For bleeding diagnosis |
| Yeh et al (2014) [24] | Public database | Ensemble of SVM-RFE^j^, neural network, and decision tree | Still-cut images | 220 bleeding images and 228 normal mucosal images | Internal test | 220 | 228 | 202 | 12 | 18 | 216 | For bleeding diagnosis (crude value of highest accuracy) |
|  |  |  |  |  |  |  |  | 206 | 18 | 14 | 210 | For bleeding diagnosis (crude value of highest sensitivity) |
| Dilna et al (2015) [40] | Public database | Histogram analysis with random forest | Still-cut images | 100 bleeding images and 100 normal mucosal images | Internal test | 100 | 100 | 98 | 1 | 2 | 99 | For bleeding diagnosis |
| Ghosh et al (2015) [41] | Public database | Statistical feature detection with SVM | Still-cut images | 200 bleeding images and 800 normal mucosal images | Internal test | 200 | 800 | 187 | 48 | 13 | 752 | For bleeding diagnosis |
| Mathew et al (2015) [42] | Unknown | Contourlet transform and Local Binary Pattern analysis with kNN | Still-cut images | 145 bleeding images and 187 normal mucosal images | Internal test | 145 | 187 | 138 | 5 | 7 | 182 | For bleeding diagnosis |
| Jia et al (2016) [43] | Unknown | CNN^k^ with SVM | Still-cut images | 2050 bleeding images and 6150 normal mucosal images | Internal test | 800 | 1000 | 794 | 1 | 6 | 999 | For bleeding diagnosis |
| Liu et al (2016) [44] | China | Joint diagonalization principal component analysis | Still-cut images | 130 bleeding images and 400 normal mucosal images | Internal test | 130 | 400 | 122 | 22 | 8 | 378 | For bleeding diagnosis |
| Yuan et al (2016) [45] | Unknown | BoW model (K-means clustering, histogram analysis) with SVM and kNN | Still-cut images |  | Internal test | 400 | 2000 | 368 | 70 | 32 | 1930 | For bleeding diagnosis |
| Jia et al (2017) [46] | Unknown | CNN | Still-cut images | 200 bleeding images and 800 normal mucosal images | Internal test | 100 | 400 | 91 | 5 | 9 | 395 | For bleeding diagnosis |
| Leenhardt et al (2019) [47] | France | CNN | Still-cut images | 300 angioectasia images and 300 normal mucosal images | Internal test | 300 | 300 | 300 | 12 | 0 | 288 | For angioectasia diagnosis |
| Aoki et al (2020) [48] | Japan | RestNet-50 | Still-cut images | 6503 bleeding images and 21344 normal mucosal images | Internal test | 208 | 10000 | 201 | 4 | 7 | 9996 | For bleeding diagnosis |
| Kundu et al (2020) [34] | Public database | Linear discriminant analysis with SVM | Still-cut images | 65 bleeding images and 1617 normal mucosal images | Internal test | 65 | 1617 | 62 | 34 | 3 | 1583 | For bleeding diagnosis |
| Tsuboi et al (2020) [49] | Japan | Single Shot Multibox Detector with CNN | Still-cut images | 2237 angioectasia images | Internal test | 493 | 10069 | 487 | 159 | 6 | 9910 | For angioectasia diagnosis |

^a^CAD: computer-aided diagnosis.

^b^TP: true positive.

^c^FP: false positive.

^d^FN: false negative.

^e^TN: true negative.

^f^BoW: bag of words

^g^SVM: support vector machine.

^h^kNN: k-nearest neighbors algorithm.

^i^MLP: multilayer perceptron.

^j^RFE: recursive feature elimination.

^k^CNN: convolutional neural network.
