# Supplementary material for: Computer-Aided Diagnosis of Gastrointestinal Ulcer and Hemorrhage Using Wireless Capsule Endoscopy: Systematic Review and Diagnostic Test Accuracy Meta-analysis
Source: J Med Internet Res. 2021 Dec 14;23(12):e33267. doi: 10.2196/33267 (PMC8715364; doi:10.2196/33267)
Supplement: Multimedia Appendix 3 [file jmir_v23i12e33267_app3.docx]

**Multimedia appendix 3.** Summary of performance and subgroup analysis of the included studies for the diagnosis of ulcers or erosions in wireless capsule endoscopy images using computer-aided diagnosis.

| Subgroup | | Included studies, n | Sensitivity (95% Confidence interval) | Specificity (95% Confidence interval) | PLR^a^ (95% Confidence interval) | NLR^b^ (95% Confidence interval) | DOR^c^ (95% Confidence interval) | AUC^d^ (95% Confidence interval) |
| --- | --- | --- | --- | --- | --- | --- | --- | --- |
| All the included studies based on the standard of high accuracy | | 19 | 0.93 (0.89-0.95) | 0.92 (0.89-0.94) | 11.2 (8.6-14.7) | 0.08 (0.05-0.12) | 138 (79-243) | 0.97 (0.95-0.98) |
| All the included studies based on the standard of high sensitivity | | 19 | 0.93 (0.89-0.95) | 0.92 (0.89-0.94) | 10.9 (8.2-140.5) | 0.08 (0.05-0.12) | 135 (76-241) | 0.97 (0.95-0.98) |
| **Ethnicity of data** | | | | | | | | |
|  | Asian | 10 | 0.94 (0.90-0.96) | 0.92 (0.88-0.95) | 11.9 (8.1-17.5) | 0.07 (0.05-0.10) | 168 (101-280) | 0.98 (0.96-0.99) |
|  | Western | 3 | 0.96 (0.95-0.97) | 0.96 (0.95-0.96) | 9.7 (3.3-28.7) | 0.12 (0.02-0.62) | 78 (6-1003) | 0.98 (0.97-0.98) |
|  | Public database or unknown ethnicity | 6 | 0.92 (0.79-0.98) | 0.91 (0.87-0.94) | 10.8 (6.8-170.2) | 0.08 (0.03-0.26) | 131 (28-611) | 0.95 (0.93-0.97) |
| **Published year** | | | | | | | | |
|  | <10 years (published within 10 years) | 14 | 0.94 (0.91-0.96) | 0.92 (0.90-0.94) | 12.0 (9.1-15.7) | 0.07 (0.04-0.10) | 183 (97-346) | 0.97 (0.95-0.98) |
|  | >10 years | 5 | 0.87 (0.78-0.93) | 0.92 (0.80-0.97) | 11.4 (4.3-30.1) | 0.14 (0.08-0.24) | 82 (27-248) | 0.95 (0.92-0.96) |
| **Included images for the training data set, n** | | | | | | | | |
|  | 100≤ | 16 | 0.94 (0.91-0.96) | 0.92 (0.89-0.93) | 11.3 (8.8-14.5) | 0.07 (0.05-0.10) | 168 (97-292) | 0.97 (0.95-0.98) |
|  | <100 or unknown | 3 | 0.79 (0.71-0.85) | 0.93 (0.89-0.96) | 12.0 (1.6-88.9) | 0.24 (0.17-0.34) | 46 (6-350) | 0.80 (0.67-0.93) |
| **Included images for the test data set, n** | | | | | | | | |
|  | 100≤ | 16 | 0.93 (0.90-0.96) | 0.92 (0.90-0.94) | 12.2 (9.4-15.9) | 0.07 (0.05-0.11) | 170 (101-289) | 0.97 (0.95-0.98) |
|  | <100 | 3 | 0.85 (0.74-0.92) | 0.83 (0.72-0.90) | 4.6 (1.8-11.8) | 0.22 (0.01-0.64) | 23 (3-159) | 0.89 (0.76-0.99) |
| **Methodological quality of included studies** | | | | | | | | |
|  | High quality | 7 | 0.94 (0.91-0.96) | 0.94 (0.92-0.95) | 14.6 (11.1-19.2) | 0.06 (0.04-0.09) | 236 (118-470) | 0.98 (0.96-0.99) |
|  | Unclear or low quality | 12 | 0.91 (0.85-0.95) | 0.90 (0.86-0.93) | 9.6 (6.6-13.9) | 0.09 (0.05-0.17) | 102 (48-215) | 0.96 (0.94-0.97) |
| **Type of CAD**^e^ **models** | | | | | | | | |
|  | Neural network based | 9 | 0.94 (0.90-0.96) | 0.93 (0.90-0.96) | 14.1 (9.4-21.1) | 0.07 (0.04-0.11) | 209 (98-444) | 0.98 (0.96-0.99) |
|  | Machine learning based | 10 | 0.91 (0.84-0.95) | 0.90 (0.86-0.93) | 9.1 (6.4-12.9) | 0.10 (0.05-0.18) | 93 (42-208) | 0.95 (0.93-0.97) |
| **Type of target lesions** | | | | | | | | |
|  | Ulcers | 18 | 0.93 (0.89-0.95) | 0.92 (0.89-0.94) | 11.3 (8.5-15.2) | 0.08 (0.05-0.12) | 145 (79-264) | 0.97 (0.95-0.98) |
|  | Erosions | 1 | Null | Null | Null | Null | Null | Null |
|  | Ulcers or erosions | 1 | Null | Null | Null | Null | Null | Null |

^a^PLR: positive likelihood ratio.

^b^NLR: negative likelihood ratio.

^c^DOR: diagnostic odds ratio.

^d^AUC: area under the curve.

^e^CAD: computer-aided diagnosis.
