# Supplementary material for: Computer-Aided Diagnosis of Gastrointestinal Ulcer and Hemorrhage Using Wireless Capsule Endoscopy: Systematic Review and Diagnostic Test Accuracy Meta-analysis
Source: J Med Internet Res. 2021 Dec 14;23(12):e33267. doi: 10.2196/33267 (PMC8715364; doi:10.2196/33267)
Supplement: Multimedia Appendix 4 [file jmir_v23i12e33267_app4.docx]

**Multimedia appendix 4.** Summary of performance and subgroup analysis of the included studies for the diagnosis of bleeding in wireless capsule endoscopy images using computer-aided diagnosis.

| Subgroup | | Included studies, n | Sensitivity (95% Confidence interval) | Specificity (95% Confidence interval) | PLR^a^ (95% Confidence interval) | NLR^b^ (95% Confidence interval) | DOR^c^ (95% Confidence interval) | AUC^d^ (95% Confidence interval) |
| --- | --- | --- | --- | --- | --- | --- | --- | --- |
| All the included studies based on the standard of high accuracy | | 19 | 0.96 (0.94-0.97) | 0.97 (0.95-0.99) | 380.3 (190.6-740.8) | 0.04 (0.03-0.07) | 888 (343-2303) | 0.99 (0.98-0.99) |
| All the included studies based on the standard of high sensitivity | | 19 | 0.96 (0.94-0.97) | 0.97 (0.95-0.99) | 370.5 (190.1-730.6) | 0.04 (0.03-0.07) | 881 (339-2292) | 0.99 (0.98-0.99) |
| **Ethnicity of data** | | | | | | | | |
|  | Asian | 5 | 0.95 (0.90-0.98) | 0.98 (0.89-0.99) | 46.0 (7.7-274.3) | 0.05 (0.02-0.10) | 923 (91-9355) | 0.98 (0.97-0.99) |
|  | Public database or unknown ethnicity | 13 | 0.95 (0.93-0.97) | 0.97 (0.95-0.99) | 35.0 (17.8-68.8) | 0.05 (0.03-0.08) | 730 (268-1989) | 0.99 (0.98-0.99) |
|  | Western | 1 | Null | Null | Null | Null | Null | Null |
| **Published year** | | | | | | | | |
|  | <10 years (published within 10 years) | 15 | 0.97 (0.95-0.98) | 0.98 (0.96-0.99) | 52.1 (24.1-112.5) | 0.03 (0.02-0.05) | 1610 (572-4530) | 0.99 (0.98-0.99) |
|  | >10 years | 4 | 0.90 (0.87-0.93) | 0.92 (0.86-0.96) | 11.6 (6.5-20.7) | 0.11 (0.08-0.14) | 109 (61-195) | 0.95 (0.93-0.97) |
| **Included images for the training data set, n** | | | | | | | | |
|  | 100≤ | 15 | 0.97 (0.94-0.98) | 0.98 (0.96-0.99) | 48.3 (21.7-107.7) | 0.04 (0.02-0.06) | 1368 (445-4207) | 0.99 (0.98-0.99) |
|  | <100 or unknown | 4 | 0.92 (0.89-0.94) | 0.94 (0.88-0.98) | 16.6 (7.7-36.0) | 0.09 (0.07-0.11) | 187 (91-386) | 0.95 (0.92-0.96) |
| **Included images for the test data set, n** | | | | | | | | |
|  | 100≤ | 19 | 0.96 (0.94-0.97) | 0.97 (0.95-0.99) | 38.3 (19.6-74.8) | 0.04 (0.03-0.07) | 888 (343-2303) | 0.99 (0.98-0.99) |
|  | <100 | 0 | Null | Null | Null | Null | Null | Null |
| **Methodological quality of included studies** | | | | | | | | |
|  | High-quality | 3 | 0.99 (0.98-0.99) | 0.98 (0.97-0.98) | 29.9 (9.7-91.7) | 0.02 (0.01-0.10) | 2184 (185-25842) | 0.99 (0.98-0.99) |
|  | Unclear or low-quality | 16 | 0.95 (0.93-0.99) | 0.98 (0.95-0.99) | 41.6 (18.2-95.1) | 0.05 (0.04-0.08) | 794 (261-2411) | 0.99 (0.97-0.99) |
| **Type of CAD**^e^ **models** | | | | | | | | |
|  | Neural network-based | 8 | 0.97 (0.93-0.99) | 0.98 (0.94-0.99) | 63.3 (15.6-256.8) | 0.03 (0.01-0.07) | 2257 (332-15337) | 0.99 (0.98-0.99) |
|  | Machine learning-based | 11 | 0.94 (0.92-0.96) | 0.96 (0.94-0.97) | 23.4 (15.3-36.0) | 0.06 (0.04-0.08) | 402 (218-742) | 0.99 (0.97-0.99) |
| **Type of target lesions** | | | | | | | | |
|  | Bleeding | 17 | 0.95 (0.93-0.96) | 0.98 (0.95-0.99) | 39.1 (18.0-85.3) | 0.05 (0.04-0.08) | 738 (259-2104) | 0.98 (0.97-0.99) |
|  | Angioectasia | 2 | 0.99 (0.98-0.99) | 0.98 (0.98-0.99) | 39.5 (10.3-151.2) | 0.01 (0.01-0.04) | 5469 (2488-12025) | Null |

^a^PLR: positive likelihood ratio.

^b^NLR: negative likelihood ratio.

^c^DOR: diagnostic odds ratio.

^d^AUC: area under the curve.

^e^CAD: computer-aided diagnosis.
